# Metabolome and transcriptome profiling revealed the enhanced synthesis of volatile esters in Korla pear

**DOI:** 10.1186/s12870-023-04264-1

**Published:** 2023-05-19

**Authors:** Yuan Liu, Huan Wen, Xiaoping Yang, Cuiyun Wu, Jiaqi Ming, Hongyan Zhang, Jiajing Chen, Jiangbo Wang, Juan Xu

**Affiliations:** 1 National Key Laboratory for Germplasm Innovation & Utilization of Horticultural Crops, College of Horticulture and Forestry Sciences, Huazhong Agricultural University, Wuhan, 430070 China; 2 Sensory Evaluation and Quality Analysis Centre of Horticultural Products, Huazhong Agricultural University, Wuhan, 430070 China; 3 Institute of Fruit and Tea, Hubei Academy of Agricultural Sciences, Wuhan, 430064 China; 4 The National and Local Joint Engineering Laboratory of High Efficiency and Superior-Quality Cultivation and Fruit Deep Processing Technology of Characteristic Fruit Trees in Southern Xinjiang, College of Horticulture and Forestry, Tarim University, Alar, 843300 China; 5 Xinjiang Production and Construction Corps Key Laboratory of Biological Resources Protection and Utilization in Tarim Basin, Alar, 843300 China; 6 Ganzhou Agricultural Technology Extension Center, Ganzhou, 341000 China

**Keywords:** ‘Korla pear’ (*Pyrus sinkiangensis*), Flavor, Primary metabolites, Volatiles, Esters biosynthetic genes, OPLS-DA

## Abstract

**Background:**

Flavor contributes to the sensory quality of fruits, including taste and aroma aspects. The quality of foods is related to their flavor-associated compounds. Pear fruits have a fruity sense of smell, and esters are the main contributor of the aroma. Korla pear are well known due to its unique aroma, but the mechanism and genes related to volatile synthesis have not been fully investigated.

**Results:**

Flavor-associated compounds, including 18 primary metabolites and 144 volatiles, were characterized in maturity fruits of ten pear cultivars from five species, respectively. Based on the varied metabolites profiles, the cultivars could be grouped into species, respectively, by using orthogonal partial least squares discrimination analysis (OPLS-DA). Simultaneously, 14 volatiles were selected as biomarkers to discriminate Korla pear (*Pyrus sinkiangensis*) from others. Correlation network analysis further revealed the biosynthetic pathways of the compounds in pear cultivars. Furthermore, the volatile profile in Korla pear throughout fruit development was investigated. Aldehydes were the most abundant volatiles, while numerous esters consistently accumulated especially at the maturity stages. Combined with transcriptomic and metabolic analysis, *Ps5LOXL*, *PsADHL*, and *PsAATL* were screened out as the key genes in ester synthesis.

**Conclusion:**

Pear species can be distinguished by their metabolic profiles. The most diversified volatiles as well as esters was found in Korla pear, in which the enhancement of lipoxygenase pathway may lead to the high level of volatile esters at maturity stages. The study will benefit the fully usage of pear germplasm resources to serve fruit flavor breeding goals.

**Supplementary Information:**

The online version contains supplementary material available at 10.1186/s12870-023-04264-1.

## Introduction

As an important commercial fruit crop, pear is cultivated in all temperature regions worldwide. According to data from the FAO, China is the country with the largest pear production of 17 million tons annually, accounting for more than 70% of the total world production. Owing to their excellent quality, pear fruits are popular among consumers and have become the third largest fruit commodity in China. The genus *Pyrus* includes at least 22 recognized species, with more than 2,000 accessions maintained in China [1–3], including the predominant cultivated species *Pyrus bretschneideri* (*P.b*), *Pyrus pyrifolia* (*P.p*), *Pyrus sinkiangensis* (*P.s*), *Pyrus ussuriensis* (*P.u*), and *Pyrus communis* (*P.c*).

Fruit flavor is one of the crucial factors affecting the sensory quality of the product. Flavor is made up of the perception of the mouth (taste, such as sweetness, acidity, and texture) and nose (aroma) [4, 5]. As a result, the characteristics of flavor-associated compounds such as aromatic volatiles, sugars, and organic acids have been investigated in many fruit species, such as citrus [6, 7], apple [8], and pear [9–12].

Zhang et al. [13] found that the total concentration of volatiles in the peels varied significantly among citrus germplasms; Ye et al. [14] also found that the composition and content in Chinese dwarf cherries varied largely with their respective genetic backgrounds. For pears, fructose and malic acid were found to be the dominant sugar and organic acid, respectively, in the eight pear cultivars [15], and the composition and quantity of aroma volatile also vary considerably depending on the cultivar species [16, 17], ripeness[10], and storage conditions [18, 19]. In particular, as esters usually send forth fruity and sweet smells in pear cultivars [5, 20], acetate esters and hexanoate esters were identified as the main contributors to pear aroma [17, 20, 21]. Furthermore, esters were defined as the dominant chemical class among metabolites detected in different pear species [17].

Volatile esters are produced mainly through the lipoxygenase (LOX) pathway and β-oxidation in pear fruits [21, 22]. Fatty acids are catalytically cleaved into short-chain aldehydes, which are further transformed into alcohols and esters [16, 23]. Related studies indicated that the production of esters was regulated by key genes such as lipoxygenase (*LOX*), alcohol dehydrogenase (*ADH*) and alcohol acetyl transferase (*AAT*) [24–30]. Nevertheless, the spatiotemporal expression of these key genes during pear growth and their corresponding functional verification are limited [24, 31].

Here, the flavor-associated metabolites in ten pear cultivars, including primary metabolites and volatile compounds, were investigated. Then, the changes in volatiles combined with transcriptomic datasets during Korla pear fruit development were analyzed to determine the key genes that regulate ester synthesis. The results will help to clarify the biosynthesis of the unique aroma as well as improve the fruit quality of Korla pear.

## Materials and methods

### Plant materials

In this study, ten pear cultivars including *P.b* ‘Xinli No.7’, *P.b* ‘Zaosu’, *P.b* ‘Xuehua’, *P.b* ‘Dangshansuli’, *P.u* ‘Anli’, *P.c* ‘Le Counte’, *P.c* ‘Red Sensatian’, *P.p* ‘Xingzang’, *P.p* ‘Wonhwang’, *P.s* ‘Korla pear’were investigated (Table S1). Among the pear cultivars, ‘Xinli No.7’ (Sample 1) was harvested from Hebei Agricultural University (Baoding, Hebei), two cultivars (Sample 7 and Sample 10) were from Institute of Fruit and Tea, Hubei Academy of Agricultural Sciences (Wuhan, Hubei), five cultivars (Sample 4–6 and Sample 8–9) were from Zhengzhou Fruit Research Institute (Zhengzhou, Henan), Korla pears were from different orchards (Sample 17–26) in Circum-Tarim Basin (Xinjiang), and all pears were harvested at their commercial maturity stages. Besides, Korla pears at six fruit developmental stages (Sample 11–16, collected at 90, 105, 120, 135, 160, 180 days after full blossom (DAFB), respectively) were from Xinjiang Production and Construction Corps Key Laboratory of Biological Resources Protection and Utilization in Tarim Basin (Akesu, Xinjiang). All samples were kept in an icebox after sweating, and immediately transferred to the laboratory within 3 days. Peel and flesh were cut separately from samples upon their arrival, then the flesh was quickly cut into cubes of 5 mm^3^. After being frozen in liquid nitrogen, all the tissues were stored at -80 ˚C until further use. All the samples were prepared in triplicate, with 6 fruits in each biological replicate.

### Chemicals and reagents

The volatile standards were purchased from Sigma Aldrich (Saint Louis, MO, USA) and Shanghai Yuanye Ltd. (Shanghai, China). Detailed information of volatile standards was showed in Table S2. Methanol (HPLC-grade) from Fisher Scientific (Fair lawn, NJ, USA), ribitol (99%), N-methyl-N-(trimethylsilyl) trifluoroacetamide (98%) and methoxamine hydrochloride (98%) from Sigma Aldrich were applied for primary metabolites determination. A standard n-paraffin mixture (C7-C40) from ANPEL Laboratory Technologies Inc. (Shanghai, China), methyl nonanoate (98%) from Sigma Aldrich and aldrich sodium chloride (99%) from Sinopharm Chemical Reagent Co., Ltd. (Shanghai, China) were for volatile compounds profiling.

### Analysis of primary metabolites by using GC-MS

Primary metabolites in flesh cubes were detected using GC-MS as previous described [5, 6] of which being the main edible parts of pear fruits. After cubes were ground into powder with liquid nitrogen, 0.30 g of powder was thoroughly mixed with methanol containing 0.02 mg·mL^− 1^ ribitol as an internal standard. The mixture was derivatized using N-methyl-N-(trimethylsilyl) trifluoroacetamide before GC-MS analysis. Then, 1 µL of each sample was injected into a gas chromatograph (Thermo Fisher Scientific, Waltham, MA, USA) equipped with a fused-silica capillary column (30 m × 0.25 mm i.d., 0.25 μm DB-5MS stationary phase). The temperature program of GC was set as follows: 100 ˚C (1 min), heated to 184 ˚C at 3 ˚C·min^− 1^, heated to 190 ˚C at 0.5 ˚C·min^− 1^ (1 min), heated to 280 ˚C at 15 ˚C·min^− 1^ (5 min), with the pulsed split injector temperature held at 230 ˚C as the split ratio was 10:1, and the carrier gas was set at a flow rate of 1.2 mL·min^− 1^. The conditions for the mass spectrometer were as follows: EI ionization source, electron energy of 70 eV; ionization temperature 260 ˚C, transfer line temperature 280 ˚C and scanning range of 40 to 650 amu.

### Volatile separation by using HS-SPME-GC-MS

As being the tissues with the richest volatiles of pear fruit, the peels were analyzed using Headspace Solid Phase Microextraction-GC-MS (HS-SPME-GC**-**MS) [5]. Samples were quickly ground into powder with liquid nitrogen. Then, 2.50 g of powder was placed in 15-mL headspace vials together with 5 mL of 30% NaCl solution containing 1 µL·L^− 1^ methyl nonanoate as the internal standard. The vials were then placed on the platform for automatic sample injection. The fibre (50/30 µm DVB/CAR on PDMS) was first aged for 5 min and then incubated with samples at 45 ˚C for 15 min, and volatiles in the headspace of the vials were collected for 15 min. The analysis was performed with gas chromatography (Thermo Fisher Scientific, Waltham, MA, USA) equipped with a TRACE TR-5 MS capillary column (30 m × 0.25 mm × 0.25 µL, Thermo Scientific, Bellefonte, PA, USA). Helium was used as the carrier gas at a constant flow rate of 1 mL·min^− 1^. The temperature program of GC was set as follows: 40 ˚C (3 min), heated to 160 ˚C at 3 ˚C·min^− 1^ (1 min), heated to 200 ˚C at 5 ˚C·min^− 1^ (1 min), and heated to 240 ˚C at 8 ˚C·min^− 1^ (1 min), with the pulsed splitless injector temperature held at 250 ˚C. The conditions for the mass spectrometer were as follows: EI ionization source, electron energy of 70 eV; ionization temperature of 230 ˚C, transfer line temperature of 230 ˚C and scanning range of 30 to 550 amu.

### Identification and quantitation of primary metabolites and volatiles

Volatile compounds were identified based on standards (Table S2), and the rest of them were performed based on the databases of NIST/EPA/NIH Mass Spectral Library (NIST 2015) in the study. The content of substances was calculated by comparison with their respective standards, and internal standards when there are no corresponding standards.

### Total RNA extraction and RNA-Seq analysis

Total RNA of Korla pear at different developmental stages was extracted from peel tissues using the RNAprep Pure Plant Plus Kit (Polysaccharides & Polyphenolics-rich) (TIANGEN, Beijing, China) with three biological replicates. RNA integrity was assessed using an Agilent 2100 bioanalyzer (Agilent, CA, USA). After the sample passed the quality inspection, two micrograms of total RNA from each sample were used to generate the sequencing libraries using a NEBNext Ultra RNA Library Prep Kit for Illumina. The constructed RNA libraries were sequenced on an Illumina HiSeq-Xten platform in paired-end 150 bp mode in Beijing Novogene Biological Information Technology Co., Ltd. (Beijing, China).

### cDNA synthesis and qRT–PCR analysis

cDNA was synthesized using HiScript II QRT SuperMix for qPCR (+ gDNA wiper) (Vazyme, Nanjing, China). One microgram of RNA was added to a 20 µL reaction mix according to the manufacturer’s instructions. All cDNA samples were diluted to a final concentration of 250 ng·µL^− 1^ with RNase-free water and then stored at -20 ˚C until use. qRT–PCR was performed with a Roche LightCycler 480 system in conjunction with Hieff qPCR SYBR Green Master Mix (Yeasen, Shanghai, China) as described by Shi et al. [32]. The primers for qRT–PCR were specifically designed based on *P.b* database gene sequences (Table S3). The *actin* sequences were used as previously described [22]. The data were analyzed by using the 2^−ΔΔCt^ analysis method. qPCR data are biological replicates (n = 3) with four replicates per experiment.

### Bioinformatics and statistical analysis

The contents of all compounds were presented as the means of three biological replicates ± standard deviation (SD). SIMCA 14.1, SAS 8.1, RStudio and Excel 2016 were used for all statistical analyses. ANOVA were taken and the significant differences were detected by the Duncan’s test (*P* < 0.05) using SAS 8.1. Supervised OPLS-DA was applied to discriminate pear species by using SIMCA 14.1. The corresponding variable importance in projection (VIP) value which represented the differences of the variables was calculated in the OPLS-DA model. Generally, it is considered that compounds with VIP value greater than 1.5 play important roles in differentiation. The expressed genes were clustered into nine groups using the Mfuzz package in R. A heatmap of ester synthesis-related genes was constructed using R based on the qRT–PCR data. Pearson correlation analysis was conducted using R.

## Results

### Primary metabolites contents varied largely in pear cultivars

Primary metabolites, including seven amino acids, seven soluble sugars, and four organic acids, were investigated in pear flesh (Sample 2–10, Sample 15 and Sample 17–26, commercial maturity stage) *via* GC-MS with precolumn derivatization (Table S4). Metabolites of ten Korla pear samples at commercial maturity (160 DAFB) were published in a previous paper. The 18 primary metabolites could be detected in almost all Korla pear samples from different regions, while their sensory qualities were considered to be different by consumers [5]. As for amino acid, the levels of *L*-proline were the highest in most pear cultivars. However, the content of *L*-aspartic acid exceeded that of *L*-proline in a few cultivars (*P < 0.05*), such as ‘Xingzang’, ‘Wonhwang’ and ‘Anli’, ranking the first free amino acid. The total amino acid content ranged from 16.98 ± 5.14 µg·g^− 1^ FW to 222.74 ± 31.41 µg·g^− 1^ FW. In general, amino acids were highly accumulated in *P.p* and *P.u* compared with other pear species.

The total content of soluble sugar in all selected samples amounting from 42.60 ± 2.01 mg·g^− 1^ FW to 69.16 ± 0.79 mg·g^− 1^ FW. For Korla pear, the average content of soluble sugar reached 66.64 ± 8.67 mg·g^− 1^ FW [5]. Fructose, sorbitol, glucose, and sucrose were the main soluble sugars in pear fruits. Fructose accounted for most of the soluble sugar, with a relative content of 44.99–66.56%, except in ‘Wonhwang’, which had sorbitol as its predominant soluble sugar, accounting for 48.21% of the total. The sucrose content in two *P.p* cultivars was generally higher than that in other pear species.

The total content of organic acid varied with a range of 0.92–4.48 mg·g^− 1^ FW in pear cultivars. Malic acid, citric acid, and quininic acid were the major organic acids in all tested pears. Most pear cultivars accumulated malic acid as the dominant organic acid except *P.u* ‘Anli’, as its citric acid content higher than that of malic acid. Typically, *P.c* has been classified as the citric acid dominant type, however, two *P.c* cultivars, ‘Le Counte’ and ‘Red Sensatian’, were classified as the malic acid dominant type in this study.

**Fig. 1:**
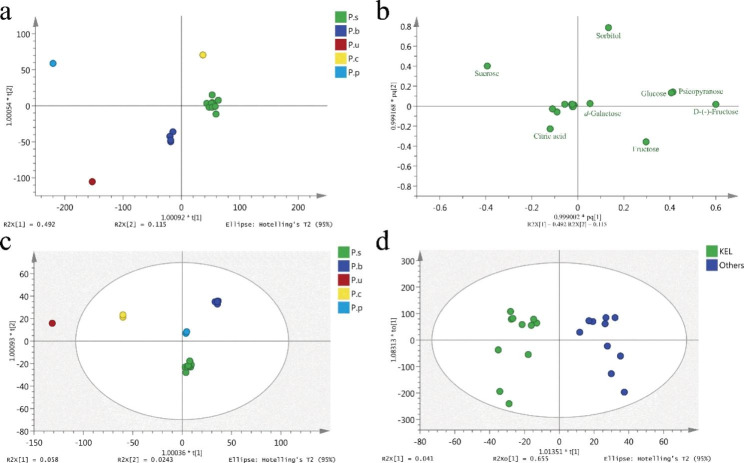
OPLS-DA of primary metabolites and volatiles in pear cultivars **a**: Score scatter plot of OPLS-DA models based on the primary metabolites with the statistical parameters (R2X = 1.000, R2Y = 0.991, Q2 = 0.574) for the classification of pear species. **b**: OPLS-DA loading plot. The materials in Fig. 1b correspond to the primary metabolites in Table S4. **c**: Score scatter plot of OPLS-DA models based on the volatiles with the statistical parameters (R2X = 0.995, R2Y = 0.993, Q2 = 0.512). **d**: Score scatter plot of OPLS-DA models based on the volatiles with the statistical parameters (R2X = 0.900, R2Y = 0.918, Q2 = 0.711). KEL: Korla pear; Others: All the pear cultivars except Korla pear

OPLS-DA was used to draw important information from the primary metabolites in pear cultivars. The score scatter plot (Sample 2–10, Sample 15 and Sample 17–26) is shown in Fig. 1a, and its corresponding loading plot is shown in Fig. 1b. Samples were clearly classified into five groups corresponding to their different species. The *P.p* group and *P.u* group were far from other pear cultivars owing to their distinctive primary metabolite profile. The loading plot helped to reveal the substances that contribute to differentiation among pear species. For example, sucrose was in the upper left corner of the second quadrant corresponding to the *P.p* group with higher sucrose content which had a similar location in the score scatter plot. Similarly, *d*-galactose and *P.s* group composed of Korla pear were all located around the centre of the scatter plot. The content of *d*-galactose in Korla pear was 206.53 ± 39.63 µg·g^− 1^ FW averagely, higher than that of other pear cultivars such as *P.u* ‘Anli’ and *P.p* ‘Wonhwang’ which were far away from the centre (Fig. 1a and b). Most amino acids and organic acids grouped together to the left of the centre, while the soluble sugars were scattered. Therefore, soluble sugars might play a more important role in distinguishing pear species than other primary metabolites.

### Fruit volatile profiles are informative to distinguish pear cultivars

Volatile compounds in all pear samples of commercial maturity stage were investigated by using HS-SPME-GC-MS. A total of 144 compounds were detected, including 17 aldehydes, 29 esters, 13 alcohols, 27 terpenes, and 58 other substances, e.g., alkanes and olefins (Table S5). The volatile constitution of pears varied greatly; eight volatile compounds were detected in all cultivars, while the other 31 compounds including 1-octen-3-one could only be detected in one cultivar. Fifty-five volatiles were detected in each sample averagely. Among them, ‘Xuehua’ contained the least number of volatiles (28), and Korla pear contained the richest compounds as 68 volatiles were detected averagely (Table 1). Esters with fruity notes were the most diverse volatile compounds of pear samples, but actually, only several esters could be detected in one pear cultivar, which means that many of them were unique. For example, Benzoic acid ethyl ester and hexyl 2-methyl-butyrate could only be detected in ‘Wonhwang’ and ‘Red Sensatian’, respectively, while only (3*Z*)-3-hexen-1-yl acetate could be detected in all pear samples. Aldehydes were the most abundant compounds. Notably, the spectrum of aldehydes in pears is relatively similar, and four aldehydes including hexanal and (*E*)-2-hexenal could be detected in all samples. These two aldehydes not only accounted for the majority of the total volatiles, but also contribute to green and grassy flavor. The total volatile content of pear cultivars ranged from 11.72 ± 0.57 µg·g^− 1^ FW to 77.35 ± 20.20 µg·g^− 1^ FW.

**Table 1:** Volatiles of pear cultivars (µg·g^− 1^ FW)

Samples^a^	Aldehydes	Esters	Alcohols	Terpenes	Others	Total
‘Xinli No.7’(HB)	43.84 ± 23.77(11)^b^	1.28 ± 0.53(4)	0.46 ± 0.16(9)	0.46 ± 0.08(6)	2.52 ± 0.24(22)	48.56 ± 23.74(52)
‘Xinli No.7’(XJ)	69.40 ± 18.90(10)	0.82 ± 0.10(4)	1.36 ± 0.43(7)	0.73 ± 0.10(6)	5.04 ± 0.79(25)	77.35 ± 20.20(52)
‘Zaosu’	16.93 ± 1.16(10)	0.45 ± 0.11(5)	0.49 ± 0.03(5)	0.26 ± 0.07(10)	1.42 ± 0.20(25)	19.55 ± 1.48(55)
‘Xuehua’	15.79 ± 1.72(4)	0.12 ± 0.06(2)	0.34 ± 0.25(6)	0.03 ± 0.02(2)	0.76 ± 0.16(14)	17.04 ± 1.96(28)
‘Dangshansuli’	14.52 ± 1.75(5)	0.44 ± 0.07(1)	0.18 ± 0.07(6)	0.41 ± 0.05(5)	1.11 ± 0.16(14)	16.67 ± 1.94(31)
‘Anli’	9.28 ± 0.58(4)	0.33 ± 0.02(1)	0.35 ± 0.22(6)	0.14 ± 0.03(5)	1.63 ± 0.08(16)	11.72 ± 0.57(32)
‘Le Counte’	9.96 ± 3.76(8)	0.63 ± 0.43(2)	0.67 ± 0.41(7)	0.59 ± 0.08(6)	2.02 ± 0.17(14)	13.87 ± 4.51(37)
‘Red Sensatian’	27.89 ± 3.80(13)	0.91 ± 0.42(2)	0.22 ± 0.10(8)	0.68 ± 0.17(3)	2.06 ± 0.12(17)	31.75 ± 4.37(43)
‘Xingzang’	47.35 ± 8.19(4)	0.97 ± 0.35(1)	0.88 ± 0.13(5)	1.62 ± 1.33(7)	1.94 ± 0.10(9)	52.76 ± 10.00(26)
‘Wonhwang’	7.50 ± 4.24(11)	3.88 ± 2.81(2)	0.28 ± 0.19(11)	0.21 ± 0.06(4)	0.94 ± 0.24(23)	12.81 ± 1.22(51)
‘Korla pear’	15.25 ± 3.90(16)	1.17 ± 0.37(9)	0.21 ± 0.03(7)	0.15 ± 0.02(10)	0.89 ± 0.21(30)	17.67 ± 3.78(72)

^a^Sample 15 at 160 DAFB was selected to represent ‘Korla pear’. ^b^The numbers tagged in the brackets represent the amount of various volatile compounds detected in pear cultivars

Different pear species varied with their aroma types, and the corresponding characteristic aroma compounds also differ greatly. OPLS-DA was used again to analyse the volatiles of pear cultivars. The score scatter plot (Sample 1–10, Sample 15 and Sample 17–26) is shown in Fig. 1c. Samples could be clearly divided into different groups corresponding to their species. *P.c* and *P.u* were the two most distant from other samples. Although Korla pear from *P.s* was located near the central area of the scatter plot, it seemed quite unique for being the only group located below the y-axis of the scatter plot.

To date, Korla pear is particularly prominent due to its pleasant fragrance and thus has created a noteworthy level of economic benefits in Xinjiang, China. To discriminate Korla pear from the other pear species, another OPLS-DA was employed, as shown in Fig. 1d. The scatter plot showed that Korla pear and other pear cultivars could be divided into two groups, indicating that the volatile profiles of Korla pear were distinctive. Those volatiles with high VIP values were considered potential biomarkers that contributed to distinguishing Korla pear from others. Here, 14 volatile compounds with VIP values greater than 1.5 were selected as volatile biomarkers, and the content differed greatly between the two groups (Table 2). The content of nonanal, 9-hexadecenoic acid, hexanoic acid and pentadecane in Korla pear was significantly higher than that in other pear cultivars, while the content of 1-hexanol, 6-methylhept-5-en-2-one and (*E*,*E*)-2,4-hexadienal in Korla pear was significantly lower. Standards of all 14 potential biomarkers were used to ensure accurate identification.

**Table 2:** List of potential biomarkers of Korla pear

No.	Compounds	VIP value	No.	Compounds	VIP value
V4	1-Hexanol	4.77	V21	Hexyl acetate	1.89
V38	Nonanal	4.30	V17	Hexanoic acid	1.83
V139	n-Hexadecanoic acid	3.15	V128	Tetradecanoic acid	1.78
V16	6-Methylhept-5-en-2-one	3.11	V103	Pentadecane	1.77
V8	(*E*,*E*)-2,4-Hexadienal	3.10	V104	α-Farnesene	1.76
V7	methoxy-phenyl-Oxime	2.22	V133	Pentadecanoic acid	1.74
V137	9-Hexadecenoic acid	2.05	V49	1-Nonanol	1.72

### Correlation network analysis reflects metabolic pathways

**Fig. 2:**
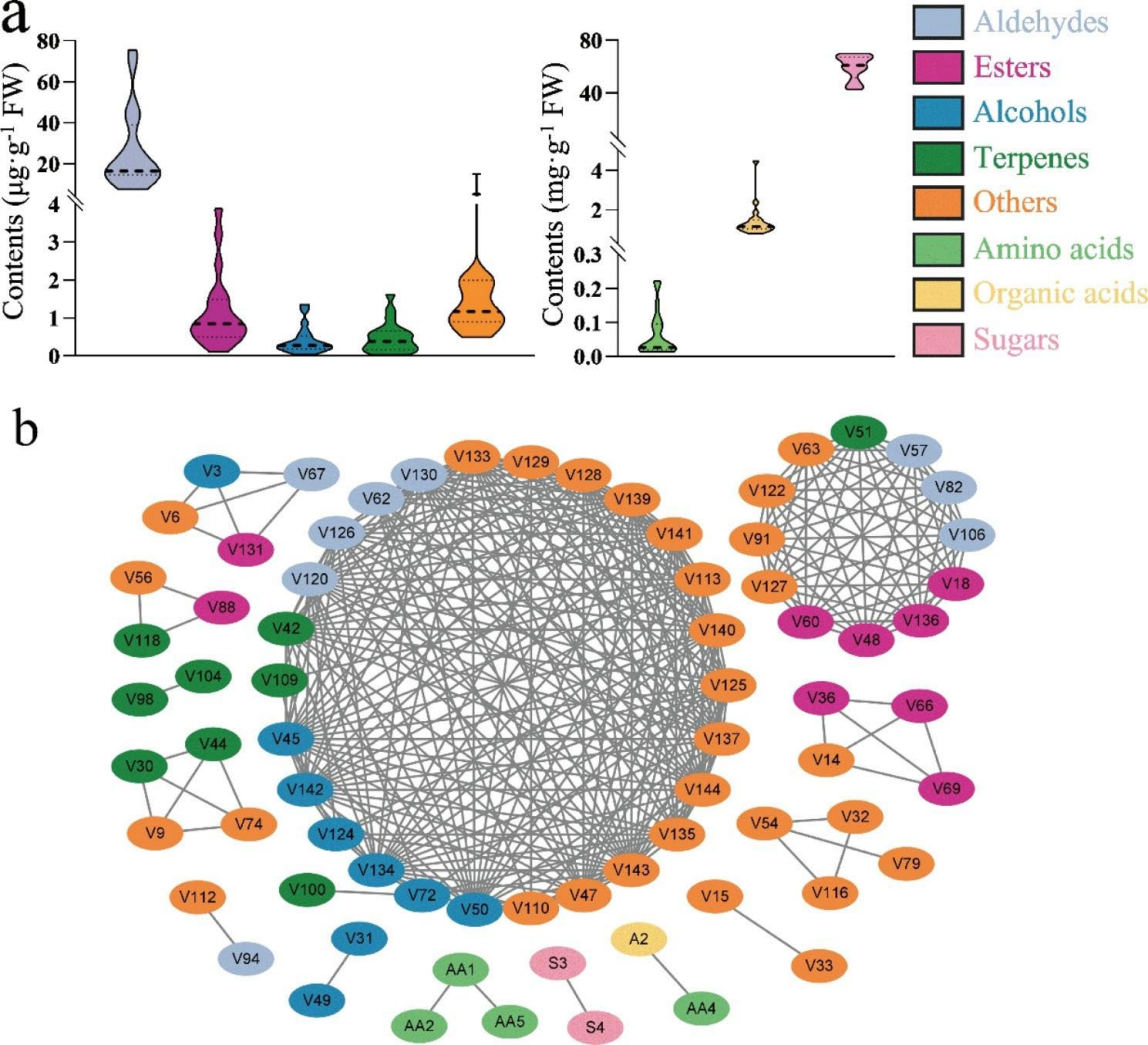
Correlation network analysis and the distribution of pear metabolites **a**: The metabolites contents of pear cultivars including primary metabolites and volatile substances. **b**: Correlation network analysis of pear cultivars based on metabolites. AA1-7 represent amino acid; A1-4 represent organic acid; S1-7 represented soluble sugar in Table S4, and V1-144 represent volatile compounds in Table S5. All correlations were at the level of r > 0.95 and *P* < 0.001

Broad variations in quantity and content of metabolites were observed for different pear cultivars (Fig. 2a). For example, the contents of the most abundant aldehydes (hexanal) in ‘Xingzang’ and ‘Le Counte’ were 26.48 ± 2.68 µg·kg^− 1^ FW and 4.92 ± 2.10 µg·kg^− 1^ FW, respectively. Qualitative differences were also observed especially in volatiles. As mentioned above, some unique substances were specifically detected only in single sample. There were the most 9 unique substances in ‘Wonhwang’ though its total volatile contents was not the highest. Correlation network analysis was performed based on the metabolite contents across all mature pear cultivars (Fig. 2b). Collinearity among substances to some extent reflected the relevance of biosynthetic pathways, helping to narrow the number of genetic targets for flavor improvement.

In the chemical networks, 73 nodes were connected by 328 significant edges. As shown, volatile substances form several large clusters, and substances with similar structures or the same metabolic pathways seem more strongly linked to each other. Sugars, organic acids and amino acids clustered into additional small clusters separately. For instance, there is a strong correlation between 1-octanol (V31) and 1-nonanol (V49) (*r* = 0.96), while *L*-Valine (AA1), *L*-Isoleucine (AA2) and *L*-Threonine (AA5) connected together in another group.

### Volatile profile changes during Korla pear fruit development

As Korla pear contains the most abundant 68 volatile substances among 10 cultivars investigated, the Korla pear fruits at six different developmental stages were used to investigate the biosynthesis of volatiles (Samples 11–16). Throughout the fruit development, totally 99 volatiles were detected, including 9 aldehydes, 19 esters, 12 alcohols, 19 terpenes and 40 other substances (Table S5). With the gradual ripening of pear fruit, more volatiles were synthesized and diffused out, and aldehydes, especially six-carbon aldehydes, were the most abundant volatiles throughout the ripening process.

**Fig. 3:**
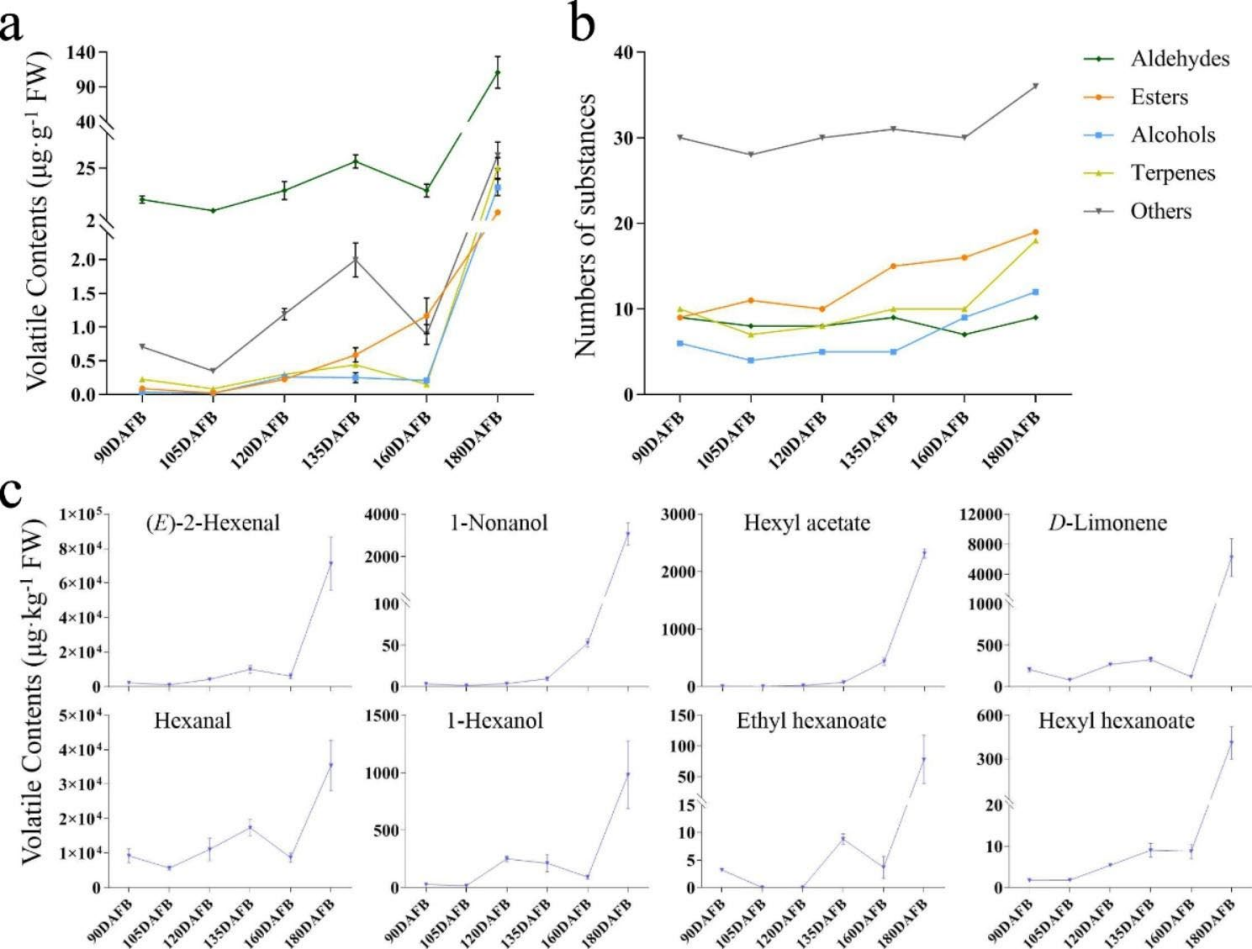
Changes of volatiles during the development of Korla pear **a**: Changes in the contents of various volatile substances during the development of Korla pear. **b**: Changes in the numbers of various volatile substances during the development of Korla pear. **c**: Changes in the contents of eight volatile substances during the development of Korla pear

Different classes of volatile showed varied expression and accumulation patterns during growth (Fig. 3a and b). There exists a clear drop around 160 DAFB in the overall upwards trend for the contents of most volatiles except esters for which increased continuously. Notably, the content of various volatiles increased sharply in the last period from 160 DAFB to 180 DAFB. In addition, several flavor substances including acetate esters, hexanoate esters, and fatty alcohols, such as hexyl acetate, hexyl hexanoate, and 1-nonanol, accumulated continuously with fruit maturation (Fig. 3c). These odorants were usually known as important flavor contributors for pear fruits as they make fruits smell floral and fruity. The odour activity values (OAVs) as well as the corresponding aroma descriptors of those odorants all confirmed their contribution to the pear flavor (Table 3).

**Table 3:** OAVs of odorants in Korla pear at different fruit developmental stages

Compounds	Aroma descriptor	Sample11	Sample12	Sample13	Sample14	Sample15	Sample16	Threshold in water (µg·kg^− 1^)
		90 DAFB	105 DAFB	120 DAFB	135 DAFB	160 DAFB	180 DAFB	
(*E*)-2-Hexenal	green, vegetable	7111	3251	13,718	33,237	20,333	237,626	0.3
Hexanal	green, grassy, fruity, orange, floral	404	248	485	763	380	1554	22.8
1-Nonanol	rose, orange	63	21	68	188	1052	61,216	0.05
1-Hexanol	fragrant, mild, sweet, green, fruity	11	6	100	85	37	392	2.5
*D*-Limonene	pungent, lemon-like	1	0	1	1	1	27	229
Hexyl acetate	fruity, apple, cherry, pear, floral	0	0	0	0	1	5	480
Ethyl hexanoate	pineapple, banana, fruity	1	0	0	2	1	16	5
Hexyl hexanoate	herbaceous	0	0	0	0	0	0	6400

### Candidate genes control esters biosynthesis in Korla pear during fruit development

The content of esters in Korla pear was at a relatively low level in the first four developmental stages and increased rapidly in the last two stages. To further investigate ester synthesis in pear fruits, the stages with low and rapid increase of ester contents were selected for sequencing analysis, respectively. Thus, the peels of Korla pear at four developmental stages of 90, 120, 160, and 180 DAFB were subjected to transcriptomic sequencing. A total of 84.13 Gb of clean base was obtained, with an average GC content of 47.07%. Then, the tag sequences were mapped to the assembled pear genome of *P.b* ‘Dangshansuli’ (Table S6). Finally, 34,367 genes were detected with various expression levels in the peels of Korla pear throughout fruit development.

**Fig. 4:**
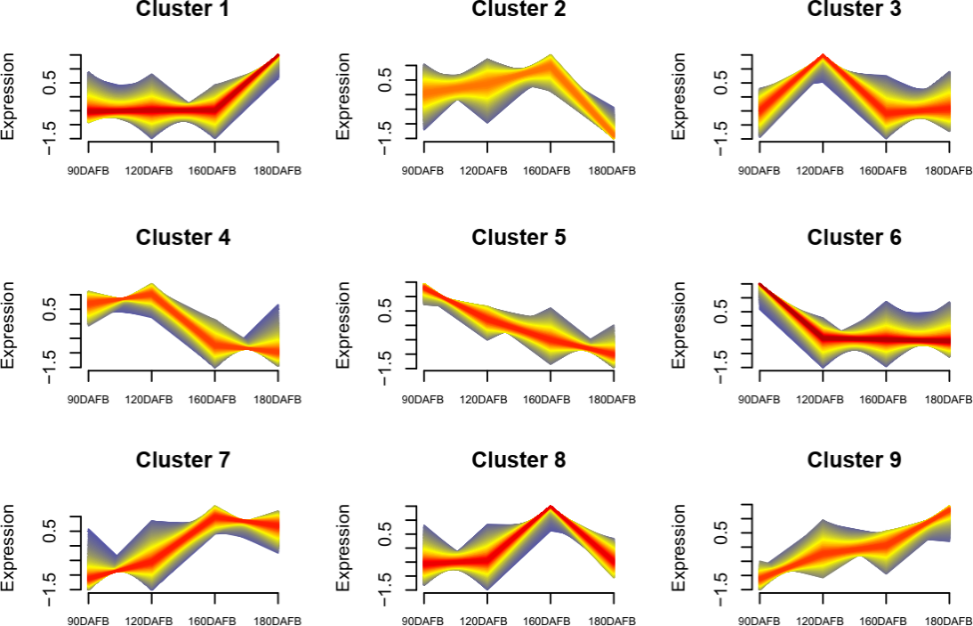
Clusters of genes having similar expression patterns during the developmental stages of Korla pear

To identify the crucial genes in the synthesis of esters, all the expressed genes were grouped using Mfuzz analysis and thus yielded nine clusters (Fig. 4, Table S7). Obviously, Clusters 1, 7, and 9 presented with the same pattern of changes to the contents of esters, while Clusters 5 and 6 showed the opposite trend.

**Fig. 5:**
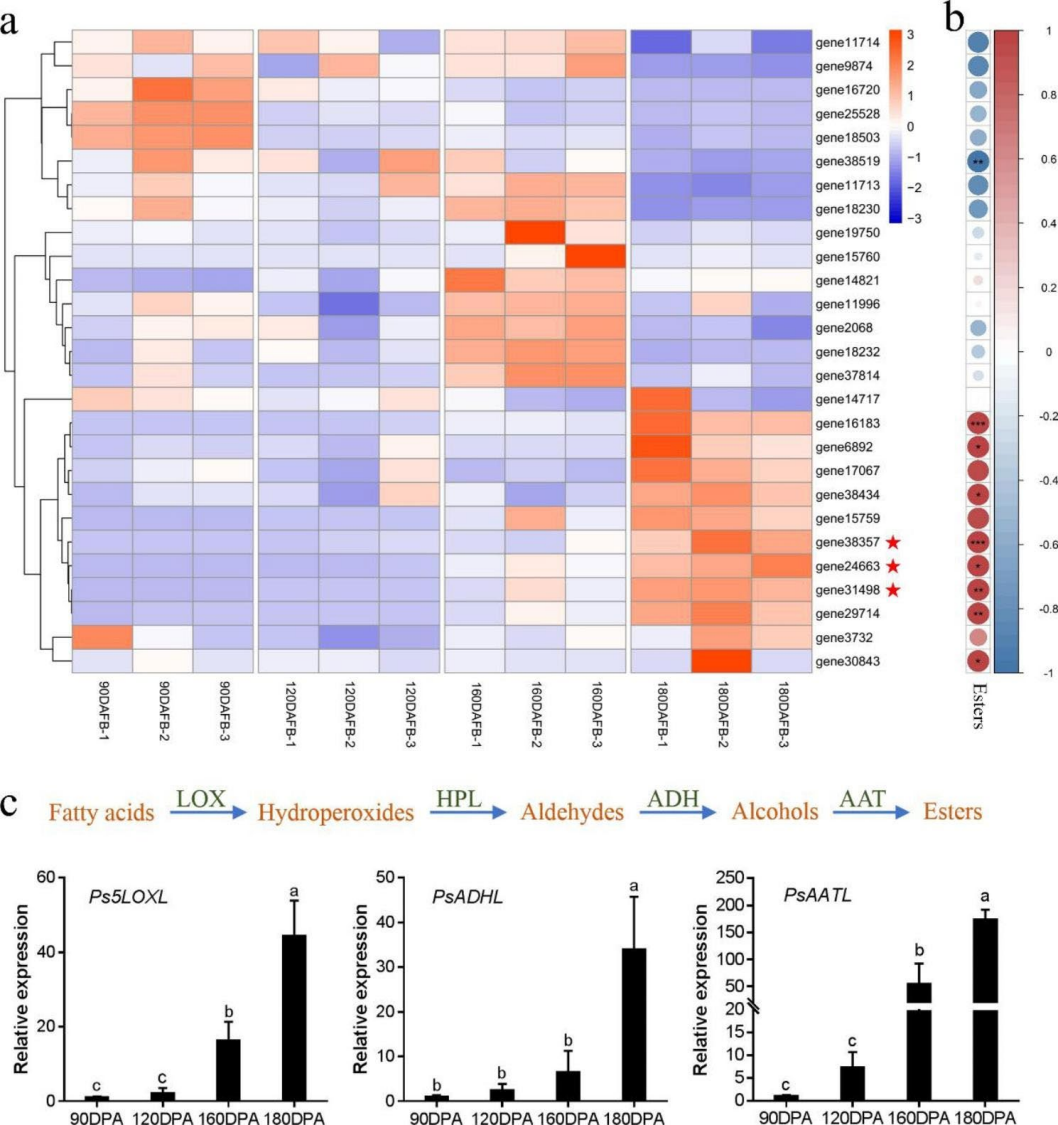
Heatmap representation of the expression levels of LOX pathway genes in Korla pear based on qRT–PCR data **a**: Relative expression levels of LOX pathway genes in Korla pear at four developmental stages, and genes marked with red stars are putative key genes. **b**: Correlation analysis of ester contents and the relative levels of LOX pathway genes in Korla pear at four developmental stages. **c**: Changes of the relative expression levels of key genes in ester synthesis

The qRT–PCR was then performed to explore the expression pattern of genes related to ester synthesis. According to the annotation of genes in the transcriptome, 27 genes were selected, and the relative expression levels are presented in a heatmap (Fig. 5a). Correlation analysis showed that eight of them were positively correlated with content of esters (*P* < 0.05) (Fig. 5b). The genes were all at low levels in the early stages, and increased continuously along with the fruit development. Lipoxygenase pathway was considered to be the main source of ester biosynthesis. The up-regulated expression of related genes like *LOX*, *ADH* and *AAT* might be the possible reason for the accumulation of ester volatiles (Fig. 5c). For such genes, the expression of *Ps5LOXL* (*gene24663*), *PsADHL* (*gene38357*), and *PsAATL* (*gene31498*) were screened out (Fig. 5c).

## Discussion

### The contribution of primary metabolites to pear flavor

For horticultural products, flavor is a vital component of sensory quality [5, 17, 30]. The pleasant flavor of fruits increases consumer preference as well as their willingness to purchase. Here, we investigated flavor-associated compounds, including soluble sugars, organic acids, amino acids, and volatiles in different pear cultivars. The metabolite spectrum varies greatly in different pear species. The results showed that fructose was the most abundant sugar in these pear fruits, followed by sorbitol and glucose, respectively. *P.p* was classified as a high sucrose type based on sugar content, while Korla pear from *P.s* was classified as a high fructose and glucose type.

Yao et al. [33, 34] evaluated the distribution of soluble sugars and organic acids in pear cultivars and then classified pears into different types especially based on soluble sugar compositions. However, grouping base on organic acid content is also practicable. In this study, most pear species were classified as the malic acid dominant type based on their organic acid content, including *P.b*, *P.p*, *P.s* and *P.c*, whilst *P.u* ‘Anli’ was the only cultivar that was classified as a citric acid dominant type. However, some *P.c* cultivars, such as ‘Bartlett’, and most *P.u* cultivars were reported to be the malic acid dominant type [33]. These two pear species generally need a post ripeness process before consumption [21, 35]. At that time, the pulp softens and usually accompanied with a rich aroma. In this paper, pears were all harvested at commercial maturity to keep fruits at comparable developmental stages. The lack of a post ripeness process was most likely the factor affecting the soluble sugar contents. In addition, the different pear varieties investigated and the metabolite determination methods may also affect the results. High levels of total organic acids result in a low sugar/acid ratio of *P.u* and *P.p*, especially *P.u* ‘Anli’ which is as low as 9.82.

Amino acids not only serve as precursors for aroma biosynthesis, but also contribute to the taste of pear fruits [15]. The major amino acids in these pear cultivars were *L*-proline and *L*-aspartic acid, which contribute to the sweetness of pears [15]. *P.p* and *P.u* contained the highest amino acid content which may have enhanced the sweetness.

### Volatiles diversify pear flavor quality

Compared to primary metabolites, volatile substances can be perceived by consumers more easily. Through comprehensive OPLS-DA analysis based on all detected metabolites, the contribution of different metabolites to the separation of pear species was obtained (Fig S1, Table S8). Volatiles were considered to have greater variation among pear species due to their high VIP values (Table S8). Therefore, volatiles might be more suitable for the separation for pear species.

To date, volatiles have been widely studied in pear [18, 25, 36–38], but few evaluations of the volatiles among different pear species have been compared [17, 39, 40]. Briefly, the distinguishing aroma differs among pear species. For example, *P.u* and *P.c* are usually described as aroma-rich types after post ripeness [11, 22, 35, 39]; whereas *P.b* and *P.p* are described as aroma-poor types; *P.s* are also classified into aroma-poor types [36]; and *P.s* ‘Korla pear’ are well known as an aroma-rich cultivar [5, 10]. In this study, 144 volatiles were detected in ten pear cultivars and the volatile profile varied considerably. Hexanal, (*E*)-2-hexenal, nonanal, 1-hexanol, *D*-limonene and hexyl acetate were detected at high levels in most cultivars, and many of them, including hexanal, (*E*)-2-hexenal, 1-hexanol, and hexyl acetate, were reported as the major pear flavor constituents [5, 10]. Korla pear contained the most numerous volatiles, and it formed a separate group in the OPLS-DA scatter plot, indicating its distinctive flavor. Furthermore, 14 volatiles, including 1-hexanol, nonanal, and n-hexadecanoic acid, were predicted as potential biomarkers of Korla pear. These results provide new insight for determining the key aroma volatiles of different pear fruits. However, sniffing and consumer preference test could be carried out to further identify the character aroma compounds that best represent the flavor of a specific pear cultivar at its consumption stage.

The aroma of pears is mainly produced in the later stages of fruit development [10, 36], so the investigation on the dynamic changes in volatiles at different developmental stages helps to illustrate their synthesis process. Chen et al. [10] found that aldehydes and alcohols exhibited continuous growth throughout three developmental stages of Korla pear. Through field investigation in Xinjiang, it was learned that although Korla pears were picked around mid-September at 160 DAFB, the pears could still keep on the tree to 180 DAFB to be full matured for a better fruity aroma. However, it is not economical neither for postharvest storage nor for transportation for fully mature pears. Based on that fact, Korla pear harvested from 90 DAFB to 180 DAFB were investigated here. As the result, a total of 99 volatiles were detected and most of the develop stages witness an increasing in volatiles contents, with the exception of 135 DAFB to 160 DAFB. In this period, the content of hexanal, (*E*)-2-hexenal and 1-hexanol all declined as they may be consumed as the precursors to hexyl esters. As known, aldehydes and olefinic aldehydes could be converted to the corresponding alcohols and further converted to esters under the catalysis of ADH and AAT, respectively. Notably, the content of hexyl acetate significantly increased in the same period (*P* < 0.01). After that, the aroma contents rose sharply from 160 DAFB to 180 DAFB, which suggested that the ripening were crucial in the formation of aroma for Korla pear.

### Ester synthesis is regulated by multiple genes

The LOX biosynthetic pathway has the potential to provide precursors for ester synthesis. LOXs, ADHs and AATs are all key enzymes in ester biosynthesis. These LOX pathway genes were reported to have a high expression level when pear fruits matured [24, 26, 28, 41]. In addition, their expression could be affected by multiple factors, such as temperature stress [18, 38]. *PbrADH6* may contribute to ester formation in pear fruit [26], and the expression of *PuADHs* was increased in glycine betaine-treated pears and thus alleviated the loss of esters in ‘Nanguo’ pear [27]. Here, three candidate genes named *Ps5LOXL*, *PsADHL*, and *PsAATL*, respectively, were found positively correlated with the ester content as their relative expression levels in the mature stages were significantly higher than those at the early stages. Given their expression pattern consistent with the level of esters, the three genes are presumed to be very likely the key genes for ester biosynthesis in Korla pear. Notably, as the last step of ester synthesis in LOX pathway, *PsAATL* showed the largest expression multiple change during the last period of pear fruit maturity in these three genes. In addition, numerous genes in the LOX pathway may also regulate esters, such as *gene 38,519* in Cluster 2, which showed a significantly negative correlation with ester content (*P* < 0.001). Furthermore, transcriptional regulation may exist, offering the potential for other genes to be explored.

## Conclusions

This study suggested that pear species can be grouped based on their metabolite profile, including soluble sugars, organic acids, amino acids, and volatile compounds. Korla pear was found to contain characteristic and the most diversified volatiles as well as esters at the commercial maturity stages of 160 DAFB among all investigated cultivars. Moreover, volatiles determination in Korla pear in different developmental stages showed that esters kept increasing continuously. Transcriptomic analysis, qRT–PCR and correlation analysis further revealed that *Ps5LOXL* (*gene24663*), *PsADHL* (*gene38357*), and *PsAATL* (*gene31498*) are hypothesised to play a key role in regulating ester biosynthesis.

## Electronic supplementary material

Below is the link to the electronic supplementary material.

Supplementary Material 1

Supplementary Material 2

Supplementary Material 3

Supplementary Material 4

## Data Availability

All data generated or analyzed during this study are included in this published article and its supplementary information files. The RNA-seq data have been deposited in the NCBI Sequence Read Archive, accession number: PRJNA925661 (https://www.ncbi.nlm.nih.gov/sra/?term=PRJNA925661).

## Abbreviations

OPLS-DA: orthogonal partial least squares discrimination analysis
DAFB: days after full blossom
FAO: Food and Agriculture Organization
LOX: lipoxygenase
ADH: alcohol dehydrogenase
AAT: alcohol acetyl transferase
GC-MS: gas chromatography-mass spectrometry
HS-SPME-GC-MS: Headspace Solid Phase Microextraction-GC-MS
EI: electron impact
qRT–PCR: quantitative real-time PCR
VIP: variable importance in projection
OAVs: odour activity values
P.s: Pyrus sinkiangensis
P.b: Pyrus bretschneideri
P.u: Pyrus ussuriensis
P.c: Pyrus communis
P.p: Pyrus pyrifolia
