# Supplementary material for: Metabolome and transcriptome profiling revealed the enhanced synthesis of volatile esters in Korla pear
Source: BMC Plant Biol. 2023 May 19;23:264. doi: 10.1186/s12870-023-04264-1 (PMC10197450; doi:10.1186/s12870-023-04264-1)
Supplement: Supplementary file 4 — Supplementary Material 4 [file 12870_2023_4264_MOESM4_ESM.docx]

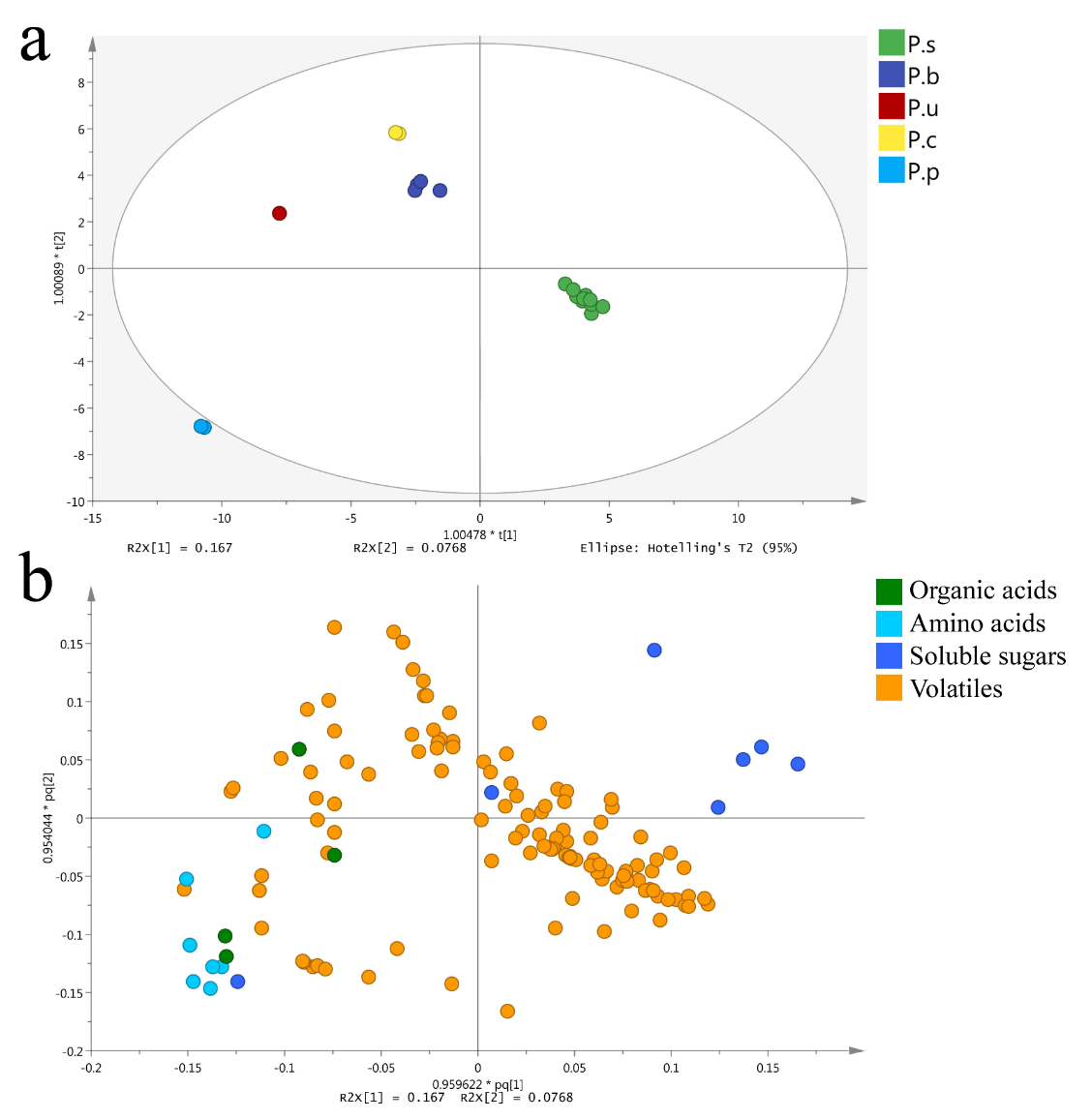


Fig. S1 OPLS-DA of metabolites in pear cultivars

a: Score scatter plot of OPLS-DA models based on the metabolites including amino acids, soluble sugars, organic acids and volatiles with the statistical parameters (R2X = 0.864, R2Y = 0.994, Q2 = 0.642) for the classification of pear species. b: OPLS-DA loading plot. The materials correspond to the metabolites in Table S4 and Table S5.
